# Variances in the Measurement of Ceramic Powder Properties

**DOI:** 10.6028/jres.100.005

**Published:** 1995

**Authors:** R. G. Munro, S. G. Malghan, S. M. Hsu

**Affiliations:** National Institute of Standards and Technology, Gaithersburg, MD 20899-0001

**Keywords:** ceramic powder, characterization, composition, density, particle size, round robin, specific surface area, variances

## Abstract

Variances in the measurement of properties used to characterize ceramic powders are discussed in the context of the International Energy Agency’s study, Annex II, Subtask 2, which includes chemical and physical measurements for five powders: two grades of silicon nitride, and one grade each of silicon carbide, silicon, and zirconia. The analysis presented here includes results for 39 properties reported by 25 laboratories using approximately 700 samples of the powders. Measurement uncertainties are discussed in the contexts of measurement variations within given laboratories (within-laboratory variance, sometimes called repeatability), among different laboratories (between-laboratory variance, also called reproducibility), and among different measurement techniques (between-methods variance). The analysis shows that the between-laboratory variance tends to be significantly greater than either the within-laboratory or the between-methods variances. The implication of this result is that the most important improvements in powder characterization measurements may be achieved through the standardization of the measurement methodologies.

## 1. Introduction

The production of most advanced ceramic materials begins with powders that are formed into useful objects or components by a variety of thermal, mechanical, and chemical processing methods. The resulting properties and performance characteristics of these materials are known to depend significantly on the chemical and physical properties of the starting powders [1]. Consequently, accurate characterization of the starting powders is essential to achieving high quality, reproducible production of current materials, and to the development of new materials with optimized or designed properties [2].

Several international efforts are making progress towards establishing measurement standards to enhance the effectiveness of powder characterization technology. The present paper discusses the measurement results reported in the International Energy Agency’s (IEA) study [3], Annex II, Subtask 2, which included chemical and physical measurements, Table 1, for five powders^1^, Table 2. The discussion uses the numerical data from the IEA study to examine the uncertainties of the powder characterization measurements when considered with respect to (A) individual laboratories (within-laboratory variance, sometimes called repeatability), (B) different laboratories (between-laboratory variance, also called reproducibility), and (C) different measurement techniques (between-methods variance).

Previous round-robin studies on ceramic powders have been hampered by sampling errors, i. e., inhomogeneities in the samples drawn from a master supply [4]. Further problems have been noted due to the degradation of the samples, especially with respect to the effects of moisture. The IEA study used painstaking riffling and packaging procedures to resolve these problems, and the effectiveness of the procedures was verified by a subsequent statistical homogeneity study.

Twenty-five laboratories from Germany, Sweden, and the United States voluntarily conducted a variety of measurements for characterizing ceramic powders. Each laboratory was supplied a set of powders in which the samples for each powder had been carefully prepared as homogeneous samples from a master supply. The laboratories were requested to derive specimens from the materials supplied to them and to conduct as many of the measurements indicated in Table 1 as would be feasible for their respective facilities. Each laboratory was to adhere to the methods and procedures normally used in its own work. This approach was designed to provide a survey of methods as well as an assessment of current practices.

Thus, the lack of standardized prescriptions for the measurement techniques was a deliberate feature of the program. It was understood that this approach would necessarily limit the extent to which a detailed statistical analysis could be used to assess the measurements or to deduce correlations among the property values. Instead, it was intended that the results from the current study would identify major sources of variability in the measurements and, perhaps, would identify needs for international standards in powder characterization.

## 2. The IEA Data Analysis

Each participant was supplied a set of samples consisting of one or more vials for each of the powders in the program. The vials were prepared with special packaging and handling procedures to ensure that all participants received comparable materials for testing. Each vial was assigned a unique code that was used to identify the sample in all reports. The participants prepared their own specimens for testing as subsamples from the original vials. The participants were requested to record all significant details regarding specimen preparations and subsequent measurement procedures, as well as the test results. The participants were also encouraged to make replicate measurements whenever feasible and to indicate whether these were performed with new specimens or with specimens that had been used previously. The resulting data were compiled and classified according to the material, the measured property, and the general method used to perform the measurement.

Overall, there were 39 different properties that could be reported for each of the approximately 700 IEA samples. Each participating laboratory performed only a selected subset of these tests, consistent with the measurements normally performed by that laboratory. As a result, for example, laboratories performing physical measurements often reported particle size results, while laboratories conducting elemental analyses usually reported results for a selected set of chemical elements.

The data obtained in this study, therefore, are useful for identifying overall, or pooled, sources of variance, but not individual contributions from distinct sources such as sampling technique, specimen preparation, apparatus, and operator performance. That level of detail will be addressed in the future when specific measurement procedures are studied. The present report discusses the pooled measures of repeatability and reproducibility.

The variability in the property values determined by a given laboratory using a specified technique can be estimated by computing the standard deviation of the replicated measurements, i.e., the estimate of repeatability is based on the within-laboratory variance. An estimate of the reproducibility of a measurement can be made by computing the standard deviation of the means obtained from different laboratories using what is nominally the same technique, i.e., the estimate of reproducibility is based on the between-laboratory variance. It should be emphasized that this estimate in particular neglects differences in specimen preparation and detailed measurement procedures. When two or more methods are used to determine a property value, an overall mean can be computed for each method; then, an estimate of the robustness of a property measurement can be made by computing the standard deviation of the overall means obtained from each of the different methods.

## 3. Results

Representative examples of the three measures of variability are presented here for both physical and chemical powder characterization measurements. Bulk density, tap density, mean particle size, and specific surface area are discussed as physical characterization measurements. For chemical characterization, three illustrative cases of quantitative elemental analysis are discussed: measurements of a major constituent (nitrogen in silicon nitride), a nonmetallic impurity (oxygen in all of the powders except zirconia), and a metallic impurity (iron in all of the powders).

### 3.1 Density

**Fig. 1:**
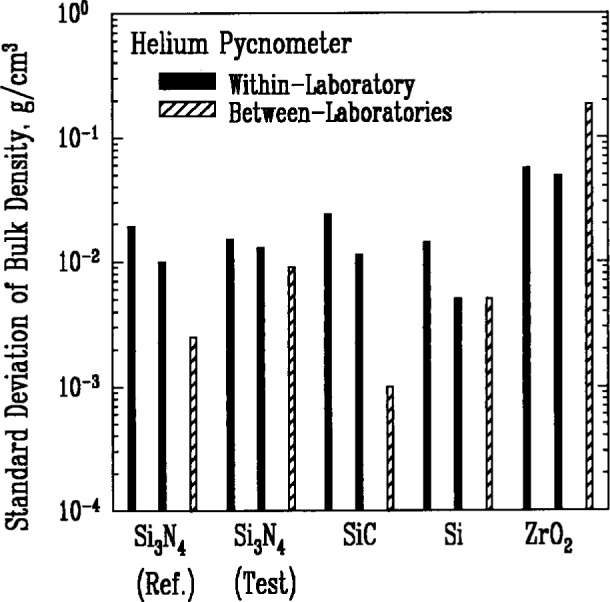
Within-laboratory and between-laboratory standard deviations in the measurements of bulk density for five powders as measured by various laboratories using the helium pycnometer method. Solid bars represent results from individual laboratories. Not all laboratories had sufficient data to calculate a meaningful standard deviation. A bar with diagonal shading represents the standard deviation of the mean values determined by the various laboratories for the indicated powder.

The bulk density of a powder refers to the average mass per unit of actual volume of the particles in the powder. The total mass of a powder specimen can be measured with high accuracy using a microbalance. The actual volume of the particles in the powder is most commonly determined by the helium pycnometer technique. The helium gas penetrates into the open pores and interparticle regions so that the volume of the gas displaced by the specimen is the sum of the volumes of the individual particles including contributions from regions of closed porosity. While only two laboratories reported measurement results, the method appears to be one of the more reproducible and reliable measurements made in this study, except for its application to zirconia. The results in Fig. 1 indicate an unusually good agreement among laboratories.

**Fig. 2:**
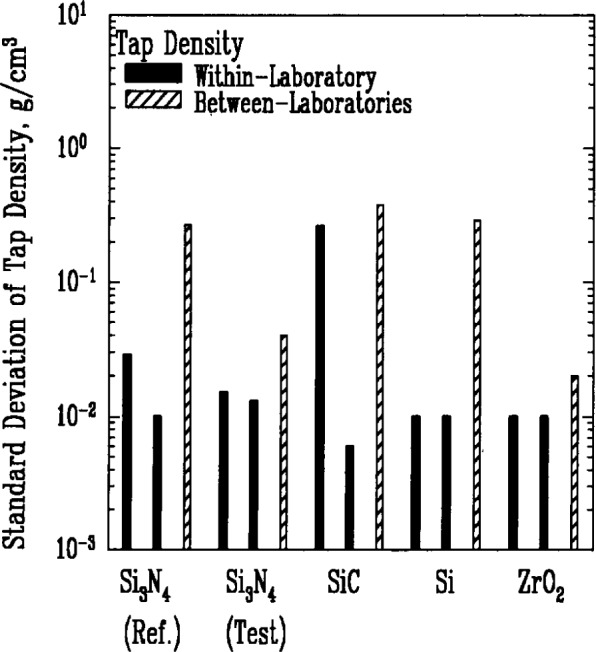
Within-laboratory and between-laboratory standard deviations in the measurements of tap density for five powders.

In contrast, the tap density measurements, Fig. 2, exhibit a much larger between-laboratory variance. This result is not surprising considering the extent to which the procedural details in this technique can influence the measured value. The tap density refers to the average mass per unit of occupied volume and is determined by dividing the total mass of the specimen by the volume occupied by the powder in a graduated cylinder after being mechanically tapped a given number of times. The powder settles or compacts as the cylinder is tapped but retains a significant pore volume. The amount of interparticle pore volume varies with both the magnitude of the impact imparted by the tapping and the frequency of the tapping.

### 3.2 Mean Particle Size

Several techniques are available to measure particle size distributions in powders. These techniques may be based on different physical principles and different instrumentation and may utilize significantly different procedures for dispersing the particles in a liquid medium before a measurement is performed. Further, the various methods have different detection limits.

Two of the methods, gravitational sedimentation and centrifugal sedimentation, are based on the size dependence of the terminal velocity of particles moving through a viscous medium under the influence of gravity or the rotational acceleration of the medium. In both methods, an equivalent spherical diameter is assumed as the measure of the particle size. Two other methods, Fraunhofer-Mie light scattering and photon correlation spectroscopy, depend on the size dependence of the distribution or diffraction pattern of the scattered light. These techniques determine a mean size of the particles in the diffraction volume.

Results from gravitational sedimentation and centrifugal sedimentation are shown in Figs. 3(a)–3(e); results for Fraunhofer-Mie light scattering are shown in Figs. 3(a)–3(d) for all powders except zirconia; and results for photon correlation spectroscopy are shown in Fig. 3(e) for zirconia. Each method measures the cumulative particle size distribution and computes the mean particle size from that distribution. In each case, the standard deviation was typically less than 10 % of the mean value, and none of the methods was consistently better than the others. The between-laboratory variance was larger than the between-methods variance for silicon nitride and silicon carbide, but for silicon and zirconia, the between-methods variances were larger.

### 3.3 Specific Surface Area

The Brunauer-Emmett-Teller (BET) gas adsorption measurement technique was used by the participants to measure the specific surface areas of the powders. There were two primary implementations of the method, denoted respectively as single point BET and multi-point BET. In the single point BET procedure, the measurement was conducted at the saturation vapor pressure of the liquified adsorbent gas. In the multi-point BET procedure, measurements were conducted at three or more adsorption pressures.

In both procedures, specimen preparation can affect the BET measurement significantly. Agglomeration of the particles and residual adsorbed gases, for example, necessarily influence the amount of adsorption that can occur during the test. Thus, sampling procedures, degassing conditions, and the choice of adsorbate gas can all be expected to contribute to variations in the results. This expectation appeared to be confirmed by the measures of variability for both methods, Figs. 4(a)–4(e), which had wide differences among the individual laboratories.

### 3.4 Major Constituents

Quantitative analysis of the major elemental components of the powders generally involved some form of decomposition of the material. Results from two methods, the combustion and Kjeldahl techniques which used chemical reactions to decompose silicon nitride, are shown in Figs. 5(a)–5(b) for the determination of nitrogen. The combustion method involves the detection and measurement of the quantity of gaseous nitrogen released from a measured mass of silicon nitride. The Kjeldahl method is a neutralization titration technique that determines the amount of nitrogen based on the amount of ammonia produced by a controlled chemical reaction.

The level of uncertainty of the Kjeldahl method was only somewhat better than the level attained by the combustion technique. The between-laboratory variances, however, were significantly different. Much better agreement between laboratories was achieved by the Kjeldahl method.

### 3.5 Nonmetallic Impurities

The major nonmetallic impurities in the powders were carbon, nitrogen, and oxygen, chlorine, fluorine, and sulfur. Two of the methods used for detecting these elements, combustion and coulometric titration, involved the decomposition of the powders in chemical reactions. A third method, fast neutron activation analysis, provided an alternative technique that did not require a decomposition reaction. Use of the latter method, however, was very limited because of the need for a nuclear reactor facility, and there were insufficient results to obtain a valid measure of its reproducibility. Results for oxygen as an impurity, Figs. 6(a)–6(d), indicated that all the methods achieved approximately the same level of variability.

### 3.6 Metallic Impurities

Atomic absorption spectroscopy (AA) and inductively coupled plasma emission spectroscopy (ICP) were used most often to determine the amount of metallic impurities in the powders. Results for iron, Figs. 7(a)–7(e), showed consistently low levels of variability within laboratories, while significant differences were often found between laboratories.

## 4. Conclusion

The most persistent trend in the results shown in Figs. 1–7 is that the between-laboratory variance tends to be greater than either the within-laboratory or between-methods variances. This trend bodes well for the effort to achieve standardization in powder characterization. The relatively small within-laboratory variances, compared with previous round-robin results, indicates the importance of the careful attention given to the primary sample preparation. By reducing the sampling error, it appears that the measurement variability within individual laboratories can, in fact, be kept relatively small for each of the measurement procedures considered in this study. Further examination of the descriptive information supplied by the participants suggests that the quantitative differences among the values measured by different laboratories often arose from varying sample pretreatment, as well as from differences in instrumentation. These sources of variability can be reduced by standardized measurement procedures. Consequently, the results presented in this study constitute a strong affirmation of the need for the standardization of powder characterization methodologies.

## Figures and Tables

**Fig. 3a:**
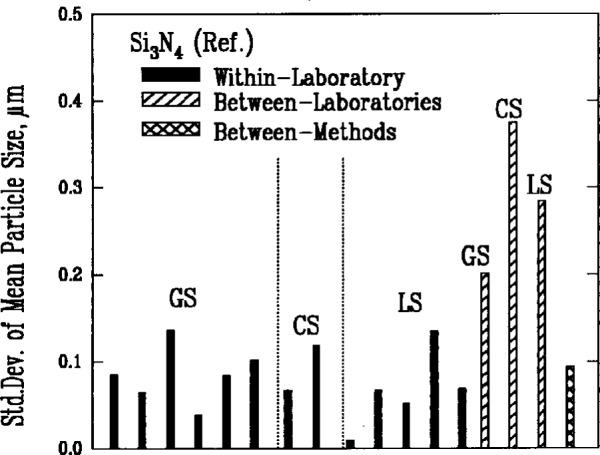
Within-laboratory, between-laboratory, and between-methods standard deviations in the measurement of particle size for the Si_3_N_4_ (Ref.) powder. The bar with cross-hatching represents the standard deviation of the mean values determined by the various methods. The methods are: GS = Gravitational Sedimentation, CS = Centrifugal Sedimentation, LS = Light Scattering.

**Fig. 3b:**
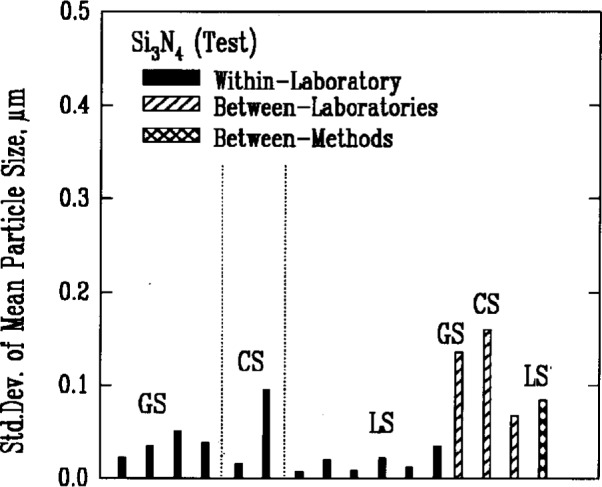
Results for the Si_3_N_4_ (Test) powder. See Fig. 3a caption for details.

**Fig. 3c:**
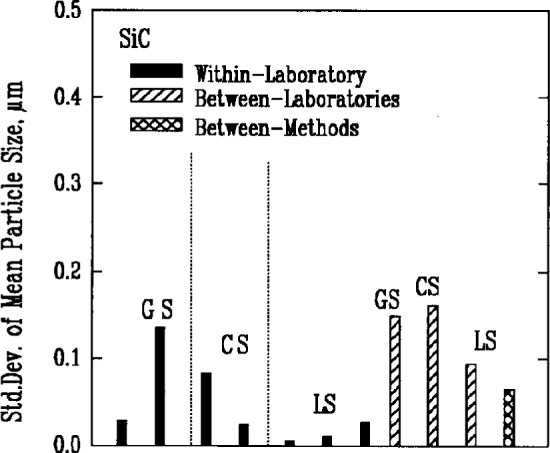
Results for the SiC powder. See Fig. 3a caption for details.

**Fig. 3d:**
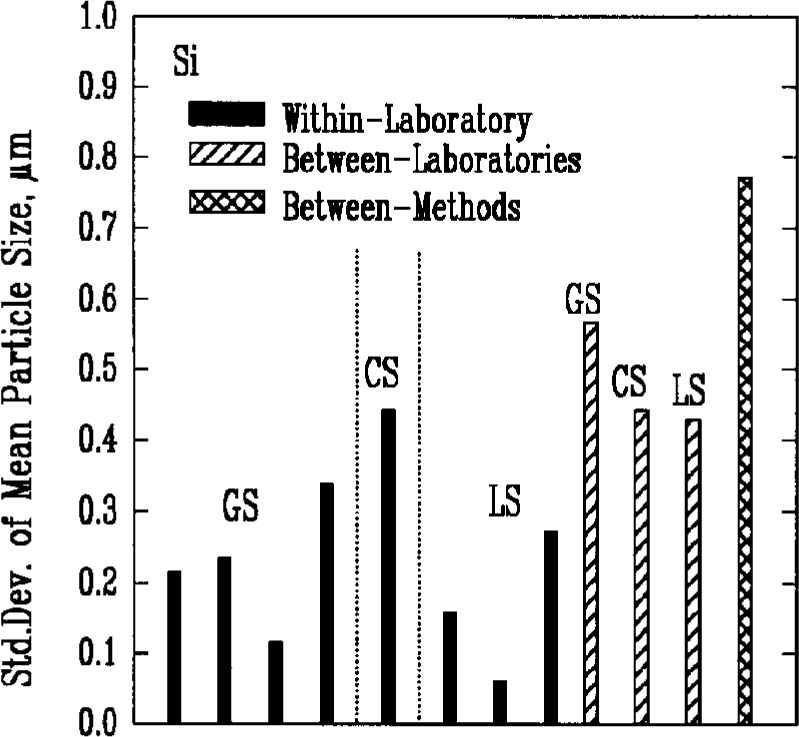
Results for the Si powder. See Fig. 3a caption for details.

**Fig. 3e:**
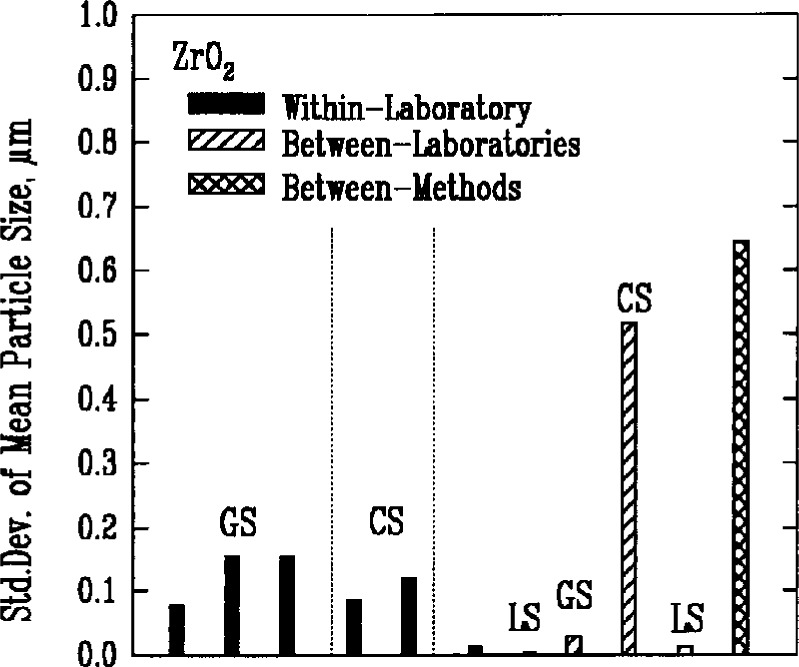
Results for the ZrO_2_ powder. See Fig. 3a caption for details.

**Fig. 4a:**
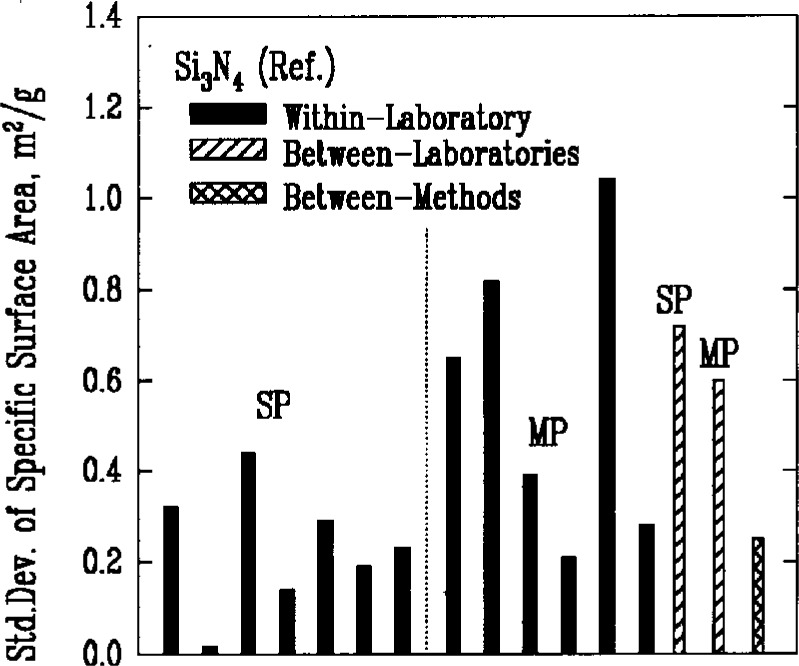
Within-laboratory, between-laboratory, and between-methods standard deviations in the measurement of specific surface area for the Si_3_N_4_ (Ref.) powder. The methods are: SP = Single Point BET, MP = Multi-Point BET.

**Fig. 4b:**
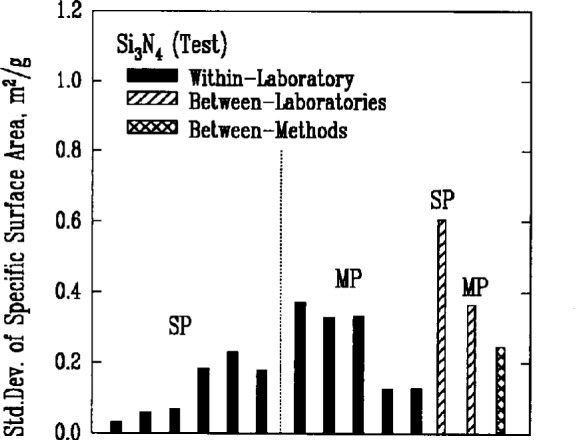
Results for the Si_3_N_4_ (Test) powder. See Fig. 4a caption for details.

**Fig. 4c:**
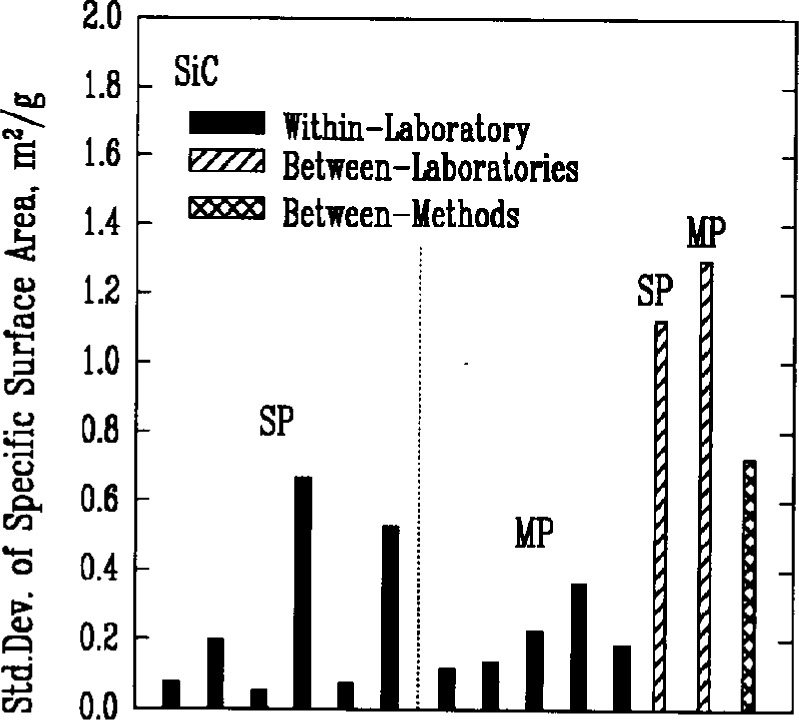
Results for the SiC powder. See Fig. 4a caption for details.

**Fig. 4d:**
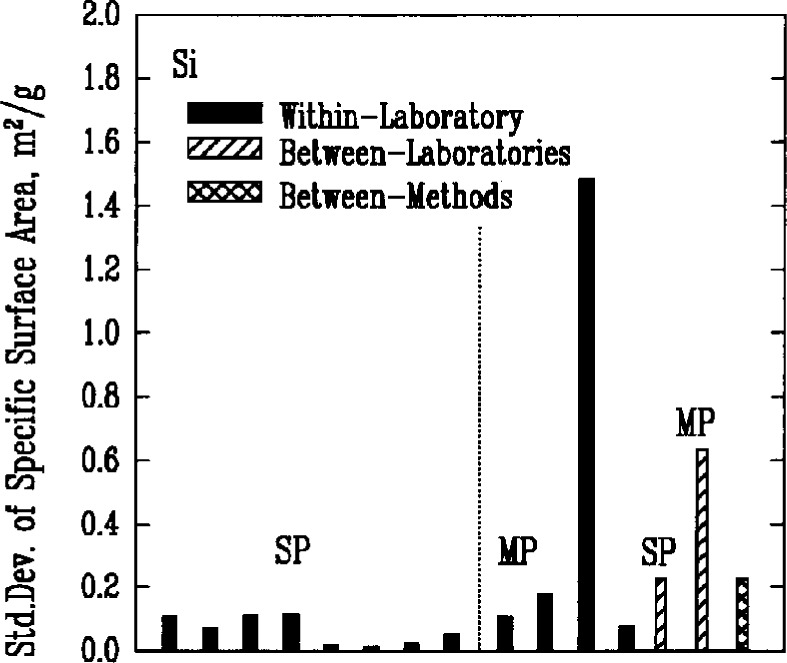
Results for the Si powder. See Fig. 4a caption for details.

**Fig. 4e:**
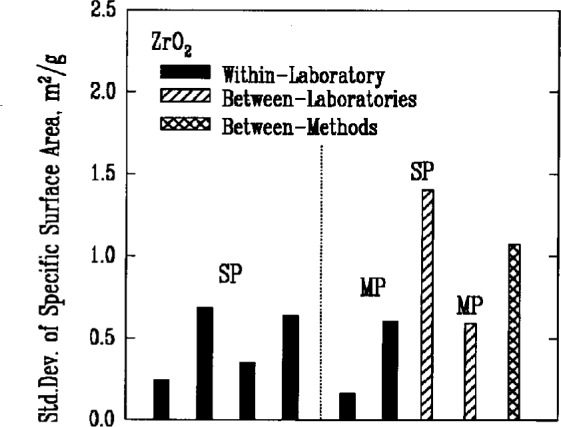
Results for the ZrO_2_ powder. See Fig. 4a caption for details.

**Fig. 5a:**
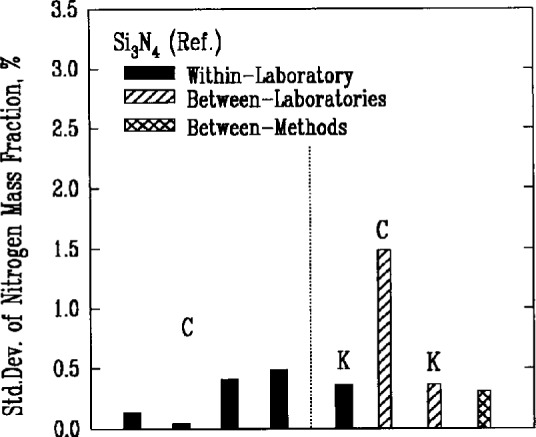
Within-laboratory, between-laboratory, and between-methods standard deviations in the measurement of nitrogen content as a major constituent in the Si_3_N_4_ (Ref.) powder. The mass fraction of the substance, in percent, is sometimes called informally the weight percent of the substance. The methods are: C = Combustion, K = Kjeldahl.

**Fig. 5b:**
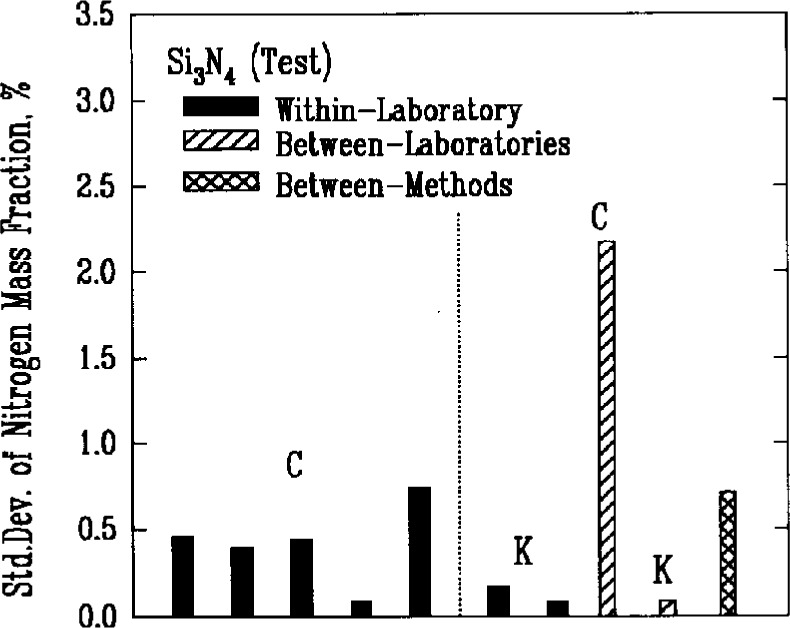
Results for the Si_3_N_4_ (Test) powder. See Fig. 5a caption for details.

**Fig. 6a:**
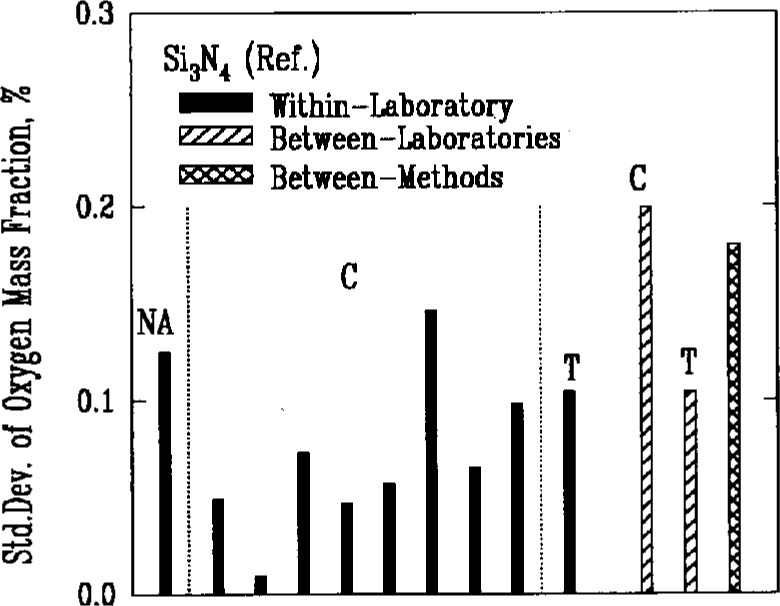
Within-laboratory, between-laboratory, and between-methods standard deviations in the measurement of oxygen content as a nonmetalic impurity in the Si_3_N_4_ (Ref.) powder. The methods are. NA = Neutron Activation, C = Combustion, T = Titration.

**Fig. 6b:**
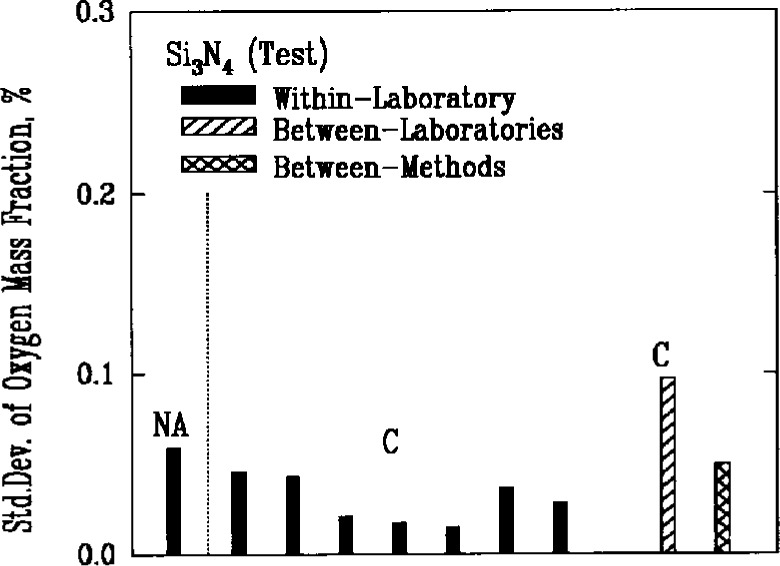
Results for the Si_3_N_4_ (Test) powder. See Fig. 6a caption for details.

**Fig. 6c:**
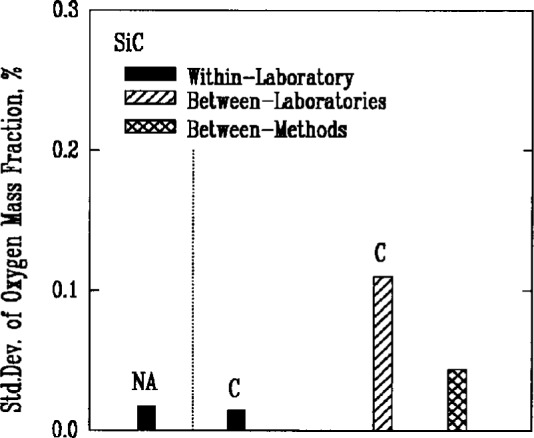
Results for the SiC powder. See Fig. 6a caption for details.

**Fig. 6d:**
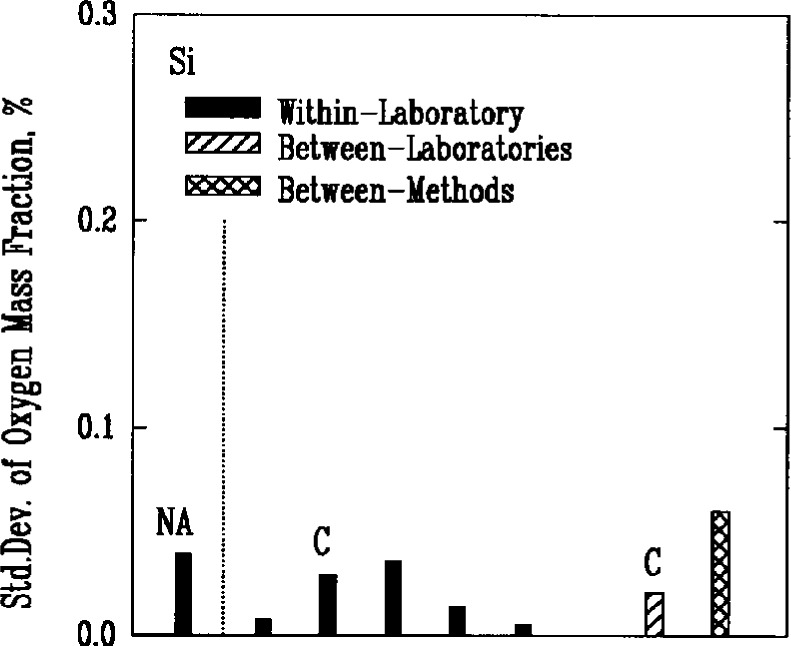
Results for the Si powder. See Fig. 6a caption for details.

**Fig. 7a:**
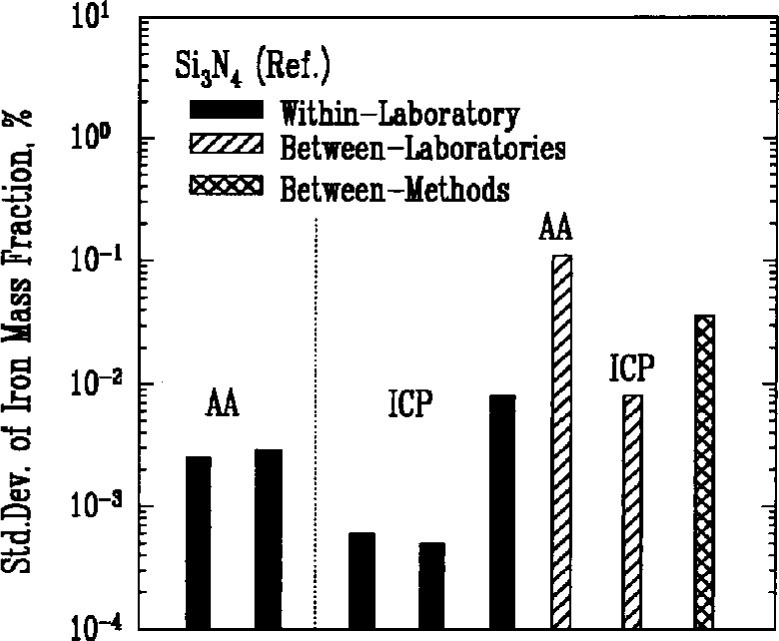
Within-laboratory, between-laboratory, and between-methods standard deviations in the measurement of iron content as a metalic impurity in the Si_3_N_4_ (Ref.) powder. The methods are: AA = Atomic Absorption, ICP = Inductively Coupled Plasma.

**Fig. 7b:**
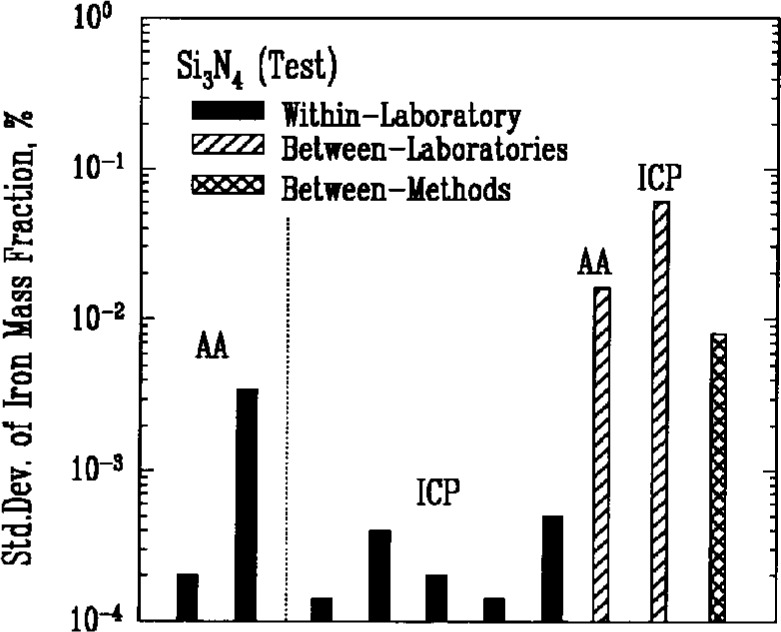
Results for the Si_3_N_4_ (Test) powder See Fig. 7a caption for details.

**Fig. 7c:**
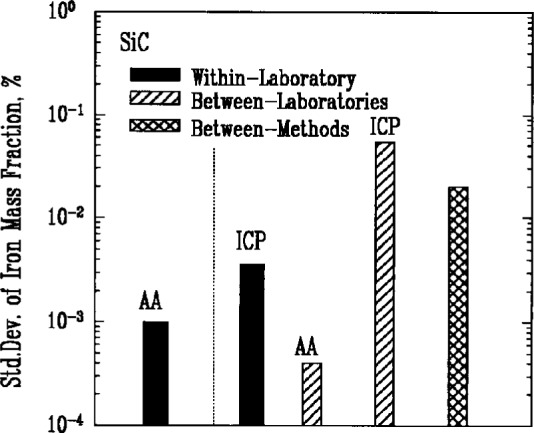
Results for the SiC powder. See Fig. 7a caption for details.

**Fig. 7d:**
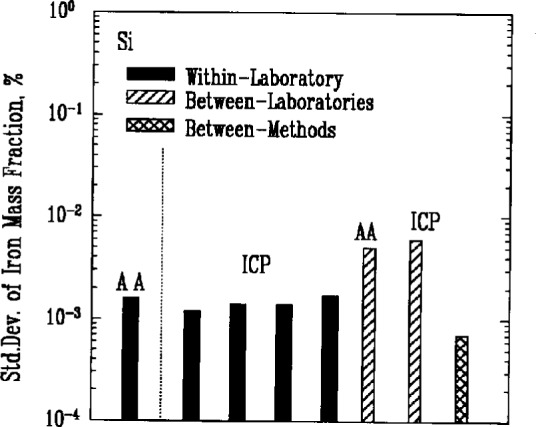
Results for the Si powder. See fig. 7a caption for details.

**Fig. 7e:**
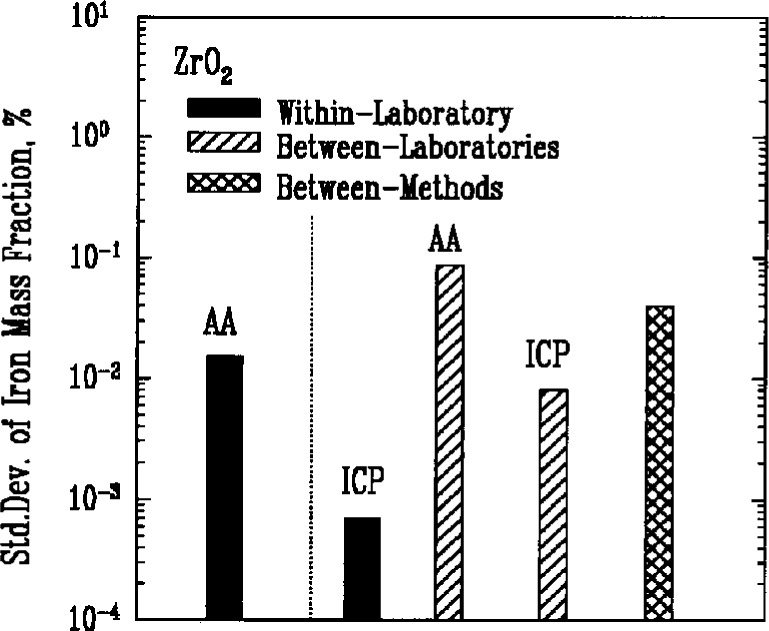
Results for the ZrO_2_ powder. See Fig. 7a caption for details.

**Table 1:** Summary of the properties measured in the IEA powder characterization program

Category	Properties
Physical properties	Density, specific surface area, particle size distribution
Major elements	Carbon, nitrogen, silicon, zirconium
Nonmetallic impurities	Chlorine, fluorine, oxygen, sulfur
Metallic impurities	Aluminum, barium, boron, calcium, free carbon, total carbon, chromium, gallium, iron, magnesium, manganese, nickel, potassium, free silicon, sodium, tin, titanium, tungsten
Other impurities	Hafnium, holmium, neodymium, yttrium

**Table 2:** The powders used in the IEA Annex II, Subtask 2, powder characterization program

Generic name	Commercial designation	Comments
Silicon nitride	H. C. Starck, LC-10	Denoted Si_3_N_4_ (Ref.)
Silicon nitride	Ube, SNE-10	Denoted Si_3_N_4_ (Test)
Silicon carbide	H. C. Starck	Particle sizes < 5 μm
Silicon	Kemanord, IV-D	Particle sizes < 10 μm
Zirconia	TOSOH	yttria stabilized, spray dried

## Footnotes

1 Certain commercial equipment, instruments, or materials are identified in this paper to foster understanding. Such identification does not imply recommendation or endorsement by the National Institute of Standards and Technology, nor does it imply that the materials or equipment identified are necessarily the best available for the purpose.

## References

[1] Veale CR (1972). Fine Powders Preparation, Properties and Uses.

[2] Schubert H, Petzow G, Somiya S (1990). Preparation and Characterization of Ceramic Powders. Advanced Ceramics III.

[b3-j10mun] 3The IEA program on powder characterization is part of an international agreement, entitled the Implementing Agreement for a Programme of Research and Development of High Temperature Materials for Automotive Engines, which is administered in the United States by the U.S. Department of Energy.

[4] Allen T (1981). Particle Size Measurement.

[5] Mandel J (1991). Evaluation and Control of Measurements.

